# A New Method to Define the VI-Ts Diagram Using Subpixel Vegetation and Soil Information: A Case Study over a Semiarid Agricultural Region in the North China Plain

**DOI:** 10.3390/s8106260

**Published:** 2008-10-07

**Authors:** Zhigang Sun, Qinxue Wang, Bunkei Matsushita, Takehiko Fukushima, Zhu Ouyang, Masataka Watanabe

**Affiliations:** 1 National Institute for Environmental Studies, 16-2 Onogawa, Tsukuba, Ibaraki 305-8506, Japan; EMails: sun.zhigang@nies.go.jp; wangqx@nies.go.jp; 2 Graduate School of Life and Environmental Sciences, University of Tsukuba, 1-1-1 Tennodai, Tsukuba, Ibaraki 305-8571, Japan; E-Mails: mbunkei@sakura.cc.tsukuba.ac.jp; fukusima@sakura.cc.tsukuba.ac.jp; 3 Institute of Geographical Sciences and Natural Resource Research, Chinese Academy of Sciences, Datun Road 11A, Beijing 100101, P. R. China; E-Mail: ouyz@igsnrr.ac.cn; 4 Faculty of Environmental Information, Keio University, 5522 Endo, Fujisawa, Kanagawa 252-8520, Japan; E-Mail: masawata@sfc.keio.ac.jp

**Keywords:** Component surface temperature, surface temperature, vegetation index, VI-Ts diagram

## Abstract

The VI-Ts diagram determined by the scatter points of the vegetation index (VI) and surface temperature (Ts) has been widely applied in land surface studies. In the VI-Ts diagram, dry point is defined as a pixel with maximum Ts and minimum VI, while wet point is defined as a pixel with minimum Ts and maximum VI. If both dry and wet points can be obtained simultaneously, a triangular VI-Ts diagram can be readily defined. However, traditional methods cannot define an ideal VI-Ts diagram if there are no full ranges of land surface moisture and VI, such as during rainy season or in a period with a narrow VI range. In this study, a new method was proposed to define the VI-Ts diagram based on the subpixel vegetation and soil information, which was independent of the full ranges of land surface moisture and VI. In this method, a simple approach was firstly proposed to decompose Ts of a given pixel into two components, the surface temperatures of soil (*T_soil_*) and vegetation (*T_veg_*), by means of Ts and VI information of neighboring pixels. The minimum *T_veg_* and maximum *T_soil_* were then used to determine the wet and dry points respectively within a given sampling window. This method was tested over a 30 km × 30 km semiarid agricultural area in the North China Plain through 2003 using Advanced Spaceborne Thermal Emission Reflection Radiometer (ASTER) and MODerate-resolution Imaging Spectroradiometer (MODIS) data. The wet and dry points obtained from our proposed method and from a traditional method were compared with those obtained from ground data within the sampling window with the 30 km × 30 km size. Results show that *T_soil_* and *T_veg_* can be obtained with acceptable accuracies, and that our proposed method can define reasonable VI-Ts diagrams over a semiarid agricultural region throughout the whole year, even for both cases of rainy season and narrow range of VI.

## 1. Introduction

**Figure 1.**
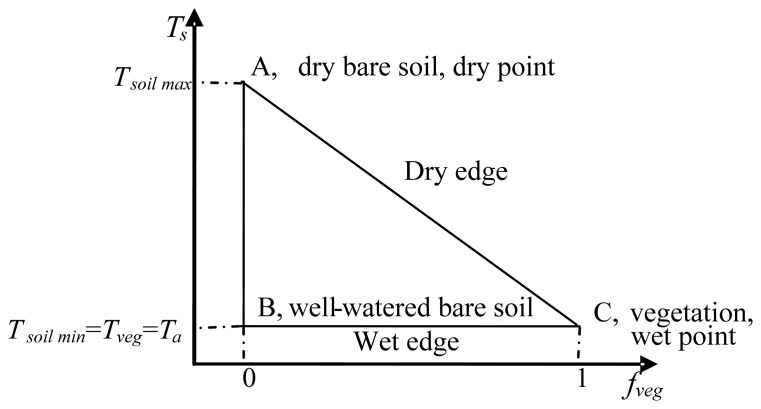
The concept of a triangular VI-Ts diagram. Point A is called a dry point, and Point C is called a wet point in the VI-Ts diagram. AC is named the dry edge, and BC is named the wet edge.

The VI-Ts diagram determined by the scatter points of remotely sensed vegetation index (VI) and land surface temperature (Ts) has been widely used to retrieve information on the partitioning of available surface energy [1-3] and surface moisture status [4-9]. All these applications are under the condition of homogenous atmospheric forcing such as solar radiation and air temperature over a sampling window for defining a VI-Ts diagram, so the size of the sampling window cannot be too large. If the sampling window covers full ranges of land surface moisture (from dry to well-watered) and VI (from bare soil to closed canopy), the VI-Ts diagram typically represents a right triangle when canopy temperature is assumed to be equal to Ts of well-watered bare soil [2, 8, 10] (Figure 1). The triangular VI-Ts diagram has been widely applied in previous studies [2-5]. The key point in these applications is how to define an ideal VI-Ts diagram, while the key point in the definition of the VI-Ts diagram is how to determine a dry edge in the VI-Ts diagram. Two automatic methods were proposed to define the dry edge in previous studies [5, 9, 11]. However, these two traditional methods require enough pixels that cover full ranges of land surface moisture and VI. Practically, it is difficult to find enough ideal pixels within a limited sampling window, especially when using a satellite data with moderate / coarse resolution, such as 1 km MODerate-resolution Imaging Spectroradiometer (MODIS) data. Generally, a natural land surface at the 1 km scale is usually a mixture of vegetation and non-vegetation (water and soil). Since water and soil surfaces have similar properties of low VI value, water surface can be considered as soil surface saturated by water. Therefore, if surface temperature (Ts) of a mixture pixel can be separated as the component surface temperatures of soil and vegetation (*T_soil_* and *T_veg_*, defined as subpixel information in this paper), two extreme surface conditions, dry bare soil (dry point) and closed vegetation (wet point), will probably be easily found at the subpixel scale. From Figure 1, the VI-Ts diagram will be readily defined if the dry and wet points are determined, which is independent of full ranges of land surface moisture and VI.

There are some available methods to estimate component surface temperatures in previous studies. Assuming that a ‘mixed’ pixel was composed of only two temperature fields, a ‘target’ temperature and a ‘background’ temperature, the surface temperatures of both ‘target’ and ‘background’ were determined using two equations based on the land surface thermal radiative energy balance of channels 3 and 4 of NOAA-6/AVHRR [12]. This multi-channel method needs the condition of the same spectral response, which is difficult to meet actually. A Bayesian-based method was proposed to estimate cloud-top subpixel brightness temperature using multi-channel infrared GOES radiometer data [13]. This method used a detailed optical model of the GOES multi-channel imaging system, which is complicated and difficult in practical applications. A genetic inverse algorithm and multi-angle thermal infrared data were used to retrieve the component temperatures of mixed pixels [14]. However, there are almost no sensors onboard the existing satellites to provide multi-angle information except the Along Track Scanning Radiometer (ATSR) launched by European Space Agency. The morning/noon data was used to retrieve thermal inertia information, and then obtain component temperatures [15]. This thermal inertia method depended on ground experiments. Because of the limitations of the above methods, some researchers were inclined to estimate the component temperatures by means of the remotely sensed vegetation index. A linear two-source model was developed to estimate evaporation fraction where soil surface temperature within a pixel was obtained using a VI-Ts diagram [2], however vegetation surface temperature within a sampling window for defining the VI-Ts diagram was assumed to be homogeneous. In this study, a new practical approach was developed to obtain the component surface temperatures (vegetation / soil) based on the spatial autocorrelation of land surface moisture [16-18], in which the variation of vegetation surface temperature due to the difference of water deficit was considered.

The MODIS and the Advanced Spaceborne Thermal Emission Reflection Radiometer (ASTER) are onboard the NASA's Earth Observation System (EOS)-Terra satellite launched in 1999, both of which can provide high quality observations of land surface. MODIS was designed to collect observational data over a wide range at moderate resolutions (250, 500 and 1,000 m) with almost daily coverage of the Earth (http://modis.gsfc.nasa.gov/). ASTER captures high spatial resolution data in 14 bands, from the visible (15 m) to the thermal infrared (90 m) wavelengths, and provides a capability of stereo viewing (30 m) for the digital elevation model creation (http://asterweb.jpl.nasa.gov/). As the “zoom lens” for Terra, ASTER data can be used by other Terra and space-borne instruments for validation and calibration. Since both MODIS and ASTER are on the same satellite, ASTER provides an opportunity to validate MODIS observational data.

The purposes of this study were: (1) to propose a new approach to obtain *T_soil_* and *T_veg_* within a given pixel; (2) to propose a new practical method to define a VI-Ts diagram using the information of vegetation and bare soil components within pixels; (3) to validate the proposed method by using ASTER, MODIS and ground-based data; (4) to compare the proposed method with the traditional method across a semiarid agricultural region in the North China Plain through 2003.

## 2. Study Area and Data Collection

### 2.1. Study Area and Ground Data Collection

**Figure 2.**
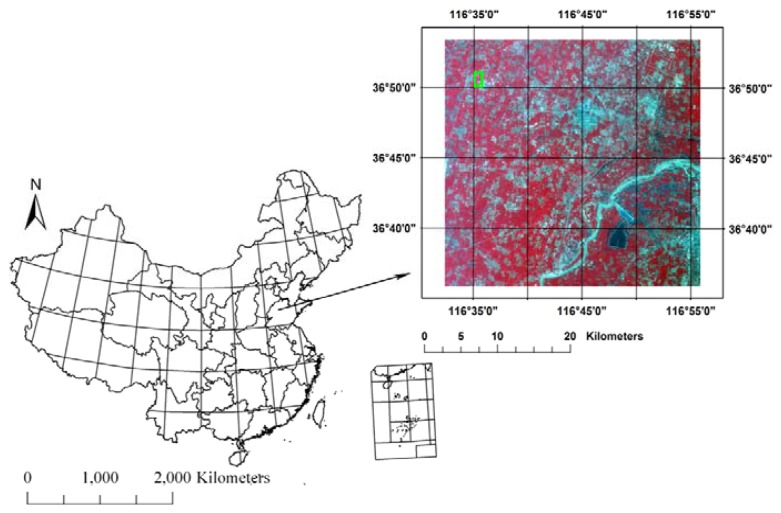
Study area. On the right is the ASTER false-color image (UTM-N50, WGS-84, 15 m, Band 3, 2, 1). The red part is vegetation, mainly winter wheat. The green rectangle in the ASTER image is the Yucheng Experimental Station (YES).

The North China Plain (NCP) is one of main crop regions in China. The region displays a typical continental monsoon climate. The yearly mean air temperature is 13.1 °C, and the annual precipitation is about 610 mm, of which about 70% falls between June and August. Therefore, the NCP is zoned as a semiarid agricultural region. Our study area (30 km × 30 km) locates at the center of the NCP (Figure 2). The light, temperature and water conditions support a 1-year 2-harvest cropping system (winter wheat (Oct.-Jun.) - summer maize (Jul.-Sep.) in this study area. Winter wheat is mainly dependent on irrigation. Usually, about four irrigations are required in the whole growing lifecycle of winter wheat. The Yucheng Experimental Station (YES, Latitude 36°49′51″ N, Longitude 116°34′18″ E, 26m above the sea level) of the Chinese Academy of Sciences locates in the study area.

Regular meteorological data recorded at the time when the EOS-Terra satellite overpassed our study area were collected from a flux station in the YES through 2003, including air temperature, humidity, wind speed, precipitation, downward and upward shortwave solar radiation, and downward and upward long-wave radiation [19]. The bulk temperature of an infinitely thick vegetation canopy is close to ambient air temperature [10], so observed air temperature can be used to validate Ts of wet points (Ts_wet, it corresponds to the minimum *T_veg_*) in this study. Assuming that the latent heat flux is 0 over the surface of dry point, the Ts of dry point (Ts_dry, it corresponds to the maximum *T_soil_*) can be obtained based on the energy balance over the dry bare soil surface [6, 20-21],

(1) $$Ts\_\text{dry}=\frac{\left(R_{n}-G)(r_{a}+r_{\text{excess}}\right)}{\rho_{a}C_{p}}+T_{a}$$

where *R_n_* and *G* are net radiation and soil heat flux in W/m^2^, *ρ_a_* and *C_p_* are the density and heat capacity of air in kg/m^3^ and J/kg/°C, *r_a_* and *r_excess_* are aerodynamic resistance and excess resistance in s/m, and *T_a_* is air temperature in °C. The methods of *r_a_* and *r_excess_* calculations are the same to those in previous literatures [6, 20, 21]. In this study, Ts_dry estimated based on ground data was used to validate those derived from remote sensing data.

### 2.2. Satellite Data Collection and Processing

**Figure 3.**
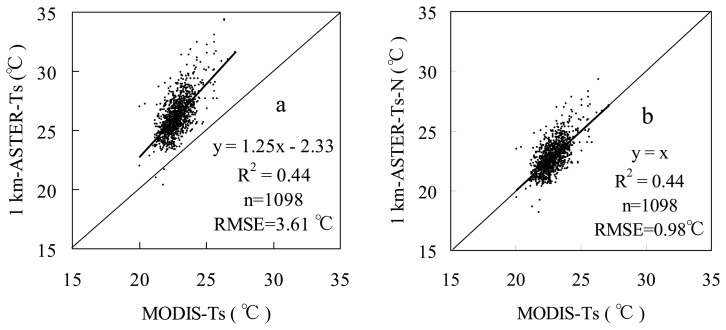
Comparisons of MODIS-Ts with (a) 1 km-ASTER-Ts and (b) normalized 1 km-ASTER-Ts.

Two level-2 data products of ASTER (projection: UTM-50N; datum: WGS-84) over our study area on May 9 2003 were collected from the Japanese Ground Data System (http://www.gds.aster. ersdac.or.jp/gds_www2002/index_e.html): AST07 (the atmospheric corrected surface reflectance, resolution: 15 m) and AST08 (the surface temperature qualitatively assessed by cloud mask, resolution: 90 m). The AST08 is produced using the Temperature Emissivity Separation (TES) algorithm that yields accuracies around 0.01 for surface emissivity and 1 °C for radiometric temperature, respectively [22]. At the same observational time of ASTER (about 11:00 am of local standard time on May 9 2003), two MODIS data products in version 5 (projection: Sinusoidal; datum: WGS-84; resolution: 1 km) were also collected from the EOS data gateway (http://edcimswww.cr.usgs.gov/pub/ imswelcome/): MOD11 (the daily land surface temperature and emissivity masked by clouds) and MOD09 (the atmospheric corrected surface reflectance). The MOD11 product has been validated in South America, and results showed that the accuracy was better than 1 °C in the range from -10 °C to 50 °C [23]. Both MODIS and ASTER products are georegistered in their making processes. The accuracy of MODIS geolocation approximates 50 m at the nadir [24]. The hand-to-hand registration accuracy of ASTER is better than 0.2 pixels [25]. The reflectances of red and near-infrared bands were used to calculate the Normalized Difference Vegetation Index (MODIS-NDVI and ASTER-NDVI). ASTER-Ts was resampled to the resolution of 1 km from 90 m by averaging pixels in order to compare the system difference between MODIS and ASTER sensors. The projection of MODIS data was transferred from Sinusoidal to UTM-50N. The statistics of above datasets were listed in Table 1. It is found that both maximum and range of MODIS-NDVI are greater than those of ASTER-NDVI, which is consistent with the report in [26]. Due to the pixel-average scaling effect [27, 28], 1 km-ASTER-Ts has a larger minimum and a smaller maximum values, and thus a narrow range compared to the 90 m-ASTER-Ts. The comparison of 1 km-ASTER-Ts with MODIS-Ts reveals that Ts observed by ASTER is a little larger than that by MODIS (Figure 3a). The root mean square error (RMSE) of the difference between 1 km-ASTER-Ts and MODIS-Ts is 3.61 °C. From Table 1, only the minimum 1 km-ASTER-Ts is close to the minimum MODIS-Ts, and other statistical items show larger differences between 1 km-ASTER-Ts and MODIS-Ts. This is caused by the difference of their respective retrieval algorithms [22-23, 27]. In order to make MODIS-Ts comparable to 1 km-ASTER-Ts, 1 km-ASTER-Ts was normalized based on their relationship in Figure 3a:

(2) 1km‐ASTER‐Ts‐N=MODIS‐Ts=(1km‐ASTER‐Ts+2.33)/1.25

where *1km-ASTER-Ts-N* is the normalized 1 km-ASTER-Ts. The RMSE of difference between the normalized 1 km-ASTER-Ts and MODIS-Ts is reduced to 0.98 °C (Figure 3b). The 90 m-ASTER-Ts was also normalized using equation (2) to remove the effects caused by the difference between their respective algorithms, and the spatial variability and scaling issues.

**Figure 4.**
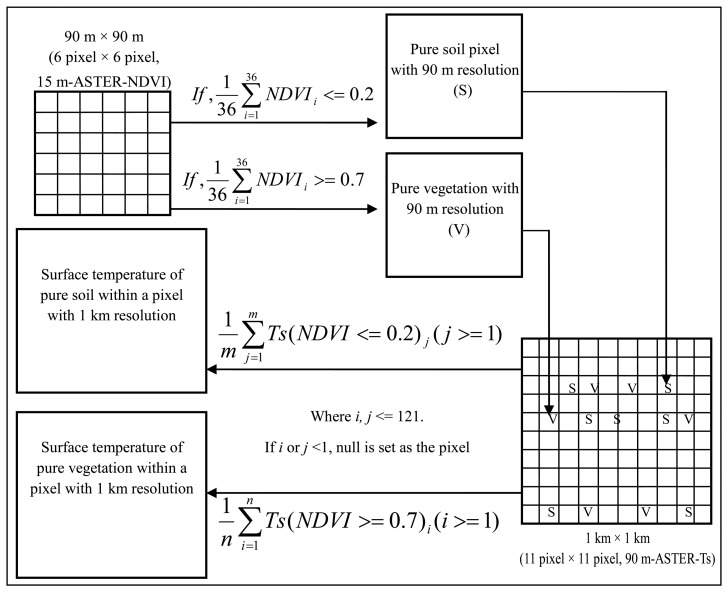
Procedure scheme of identifying the ASTER 90 m-pixels of pure soil and vegetation and obtaining Ts of pure soil and vegetation within a pixel with 1 km resolution.

In this study, MODIS-Ts was decomposed to the component Ts of vegetation and soil, and then the retrieved component Ts were evaluated by comparing with Ts of vegetation and soil retrieved from 15 m-ASTER-NDVI and normalized 90 m-ASTER-Ts. The detailed process of obtaining Ts of pure soil and vegetation was shown in Figure 4. Firstly, pure vegetation and soil pixels were identified using their respective 15 m-ASTER-NDVI thresholds, and then were resampled from 15 m to 90 m. Based on a histogram analysis and a viewing-identification on 15 m-ASTER-NDVI, it was found that NDVI of pure soil pixels was less than 0.20, while NDVI of pure vegetation pixels was greater than 0.7. These values are consistent with those proposed in a previous study [29]. Then, Ts of pure vegetation and soil pixels with 90 m resolution were averaged within a 1 km × 1 km pixel.

In order to further compare the VI-Ts diagrams defined respectively using both MODIS pixel and subpixel information, daily MODIS-Ts and MODIS-NDVI on other days through 2003 were also collected. Although only 13 cloud-free (100%) datasets were collected over our study area, these datasets could still show seasonal variations of the VI-Ts diagram (Table 2). Here, MODIS-NDVI was calculated using the MOD09 reflectance product.

## 3. Method for Estimating the Component Surface Temperatures of Vegetation and Soil

**Figure 5.**
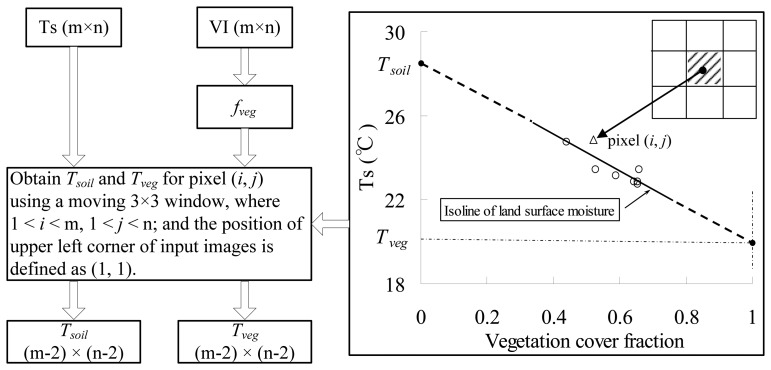
Obtaining *T*_soil_ and *T*_veg_ of a given pixel by means of 8 neighboring pixels within a 3 × 3 window. In the right figure, the moving 3 × 3 window was randomly selected in the ASTER image.

It is well known that environmental factors such as land surface moisture have a characteristic of the spatial autocorrelation [16-17]. Zhang *et al.* developed a two-layer remote sensing model for land surface flux estimations under the assumption that land surface moisture status of soil and vegetation within a pixel are the same or similar because of the same water supply sources (e.g.; from rain, irrigation, or upgoing ground water) [21]. In this study, the assumption was enlarged from one pixel to a moving window (3 pixels × 3 pixels). Thus, all these 9 pixels can be assumed to be on an isoline of land surface moisture in the VI-Ts diagram [4-9]. Therefore, *T_soil_* and *T_veg_* within this given pixel can be obtained by extending this isoline to the vertical lines of *f_veg_* = 0 and *f_veg_* = 1 (Figure 5),

(3) $$\begin{array}{l} \frac{T_{\text{soil}}-T_{\text{veg}}}{1-0}=\frac{T_{s}-T_{\text{veg}}}{1-f_{\text{veg}}}=s\\ T_{\text{veg}}=T_{s}-s(1-f_{\text{veg}})\\ T_{\text{soil}}=T_{s}+sf_{\text{veg}} \end{array}$$

where *s* is the slope of the isoline of land surface moisture, and *f_veg_* is vegetation cover fraction. It can be calculated using a second-order scaled NDVI [30-32],

(4) $$f_{\text{veg}}={\left(\frac{\text{NDVI}-{\text{NDVI}}_{\min}}{{\text{NDVI}}_{\max}-{\text{NDVI}}_{\min}}\right)}^{2}$$

where *NDVI_max_* and *NDVI_min_* are the NDVIs of full vegetation (*f_veg_* = 1) and bare soil (*f_veg_* = 0). Based on the analysis of MODIS-NDVI data during the whole growing lifecycle of winter wheat in our study area in 2003, *NDVI_max_* was given as 0.85, and *NDVI_min_* was given as 0.20. The isoline in Figure 5 can be obtained from 9 pixels by a linear regression. As for m × n Ts image, *T_soil_* and *T_veg_* images in the size of (m-2) × (n-2) can be obtained using equations (3) and (4).

Then, the size of sampling window should be determined to define the VI-Ts diagram. In this study, the size of study area is 30 km × 30 km (30 pixels × 30 pixels for 1 km-MODIS data). Hence the 28 pixels × 28 pixels area is considered as a sampling window. Within this sampling window, dry and wet points respectively correspond to soil surface with minimum moisture and vegetation surface with maximum moisture, therefore, the maximum *T_soil_* and minimum *T_veg_* can be used to determine dry and wet points, then the VI-Ts diagram can be defined only using dry and wet points. In this study, a 2^nd^ scaled NDVI was used to define the VI-Ts diagram (*f_veg_*-Ts) instead of NDVI. ASTER-Ts and ASTER-NDVI data over our study area on May 9 2003 were used to compare the NDVI-Ts, 1^st^ order NDVI-Ts and 2^nd^ order NDVI-Ts diagrams. Results show that the use of the 2^nd^ order NDVI instead of NDVI in a VI-Ts diagram does not change the triangular form.

## 4. Results

### 4.1. Obtaining MODIS-T_veg_ and MODIS-T_soil_ using the proposed approach

MODIS-T_veg_ and MODIS-T_soil_ were obtained using the approach proposed in Section 3 across the whole year of 2003. The accuracies of MODIS-T_veg_ and MODIS-T_soil_ depend on the isoline slope of land surface moisture based on the linear regression from 9 neighboring pixels. R^2^ is a measure of goodness-of-fit of linear regression. From Table 3, it is found that the means of R^2^ over the 28 pixels × 28 pixels area are all larger than 0.5 across the whole year of 2003.

### 4.2 Validations of MODIS-T_veg_ and MODIS-T_soil_ obtained using the proposed approach

#### 4.2.1 Validation using the Normalized 90 m-ASTER-Ts on May 9, 2003

Ts of pure soil and vegetation were also obtained from the normalized 90 m-ASTER-Ts according to the procedure shown in Figure 4. From Table 4, it is shown that MODIS-T_veg_ and MODIS-T_soil_ agree well with 90 m-ASTER-T_veg_-N and 90 m-ASTER-T_soil_-N, respectively. The differences of statistics between MODIS-T_veg_ and 90 m-ASTER-T_veg_-N are less than 1 °C. Compared with 90 m-ASTER-T_soil_-N, however, MODIS-T_soil_ has a narrower range because of the inherent bias between MODIS-Ts and ASTER-Ts although ASTER-Ts has been normalized. These accuracies of MODIS-T_veg_ and MODIS-T_soil_ estimations by the proposed approach are similar to those reported in [14, 33], especially for the estimation of MODIS-T_veg_.

#### 4.2.2 Validation using ground data across the whole year of 2003

**Figure 6.**
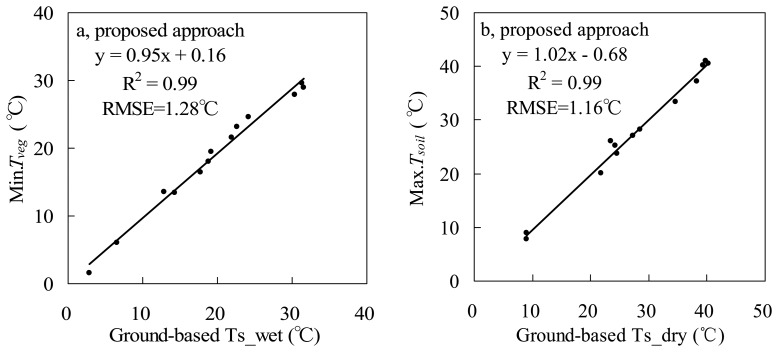
Comparisons of (a) the minimum MODIS-T_veg_ with ground-based Ts_wet, and (b) the maximum MODIS-T_soil_ with ground-based Ts_dry across the whole year of 2003.

It is hard to measure *T_soil_* and *T_veg_* at subpixel scales in fields, so it is difficult to validate retrieved *T_soil_* and *T_veg_* pixel by pixel. Another strategy was used to validate MODIS-T_veg_ and MODIS-T_soil_ obtained using the proposed approach in this study. The minimum *T_veg_* is used to determine wet point, and the maximum *T_soil_* is used to determine dry point in this study. Therefore, Ts_wet (wet point) and Ts_dry (dry point) from ground data (Section 2.1) can be reversely used to validate MODIS-T_veg_ and MODIS-T_soil_ to a certain extent. From Figure 6, results show that the minimum MODIS-T_veg_ and maximum MODIS-T_soil_ are respectively close to the ground-based Ts_wet and Ts_dry across the whole year of 2003. Both R^2^ in Figure 6 are very close to 1. Their respective RMSEs are 1.28 °C and 1.16 °C.

### 4.3 Comparisons of the Proposed and Traditional Methods for Defining the VI-Ts Diagram

#### 4.3.1 Comparisons based on the Normalized 90 m-ASTER-Ts on May 9 2003

**Figure 7.**
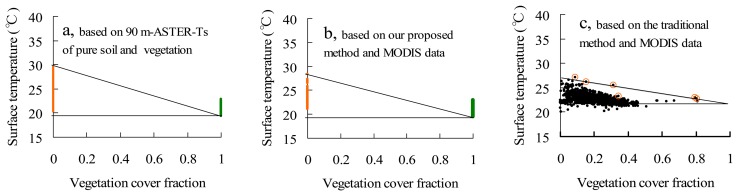
The VI-Ts diagrams on May 9 2003 (a) defined using the 90 m-ASTER-Ts of pure vegetation and soil, (b) defined by the proposed method using MODIS data, and (c) defined by the traditional method using MODIS data. In Figures 7a and 7b, yellow and green points are component Ts of soil and vegetation. In Figure 7c, yellow circles (o) are pixels for defining the dry edge.

Three triangular VI-Ts diagrams defined by different methods are shown in Figure 7. In Figure 7a, the maximum 90 m-ASTER-T_soil_-N and minimum 90 m-ASTER-T_veg_-N were used to determine dry and wet points, then to define a triangular VI-Ts diagram on May 9, 2003. This VI-Ts diagram based on the normalized 90 m-ASTER-Ts can be considered as a “true” VI-Ts diagram in this study because of its fine spatial resolution. The triangular VI-Ts diagram in Figure 7b was defined based on the information within MODIS pixels using the proposed method in this study. In Figure 7c, the triangular VI-Ts diagram was defined using the traditional method based on the information of MODIS pixels. The traditional method is a combination of two previous automatic methods [5, 9, 11]. Firstly, the pixels with maximum temperature within each small VI interval are selected in the VI-Ts scatter plot. Then, some selected pixels far from the dry edge are excluded. Finally, the dry edge is defined by a linear regression on the available selected pixels.

The VI-Ts diagram in Figure 7b is similar to that in Figure 7a, but the VI-Ts diagram in Figure 7c is far from that in Figure 7a. The wet point in Figure 7b is very close to that in Figure 7a because the difference between the minimum 90 m-ASTER-T_veg_-N and the minimum MODIS-T_veg_ is less than 0.2 °C. However, the dry point in Figure 7b is a little lower than that in Figure 7a because the Ts_dry in Figure 7b is 1.15 °C lower than that in Figure 7a. If this shift of dry point between Figures 7a and 7b resulted from the Ts bias between MODIS and ASTER is ignored, the VI-Ts diagrams are very close between two figures. However, the Ts_dry in Figure 7c (26.84 °C) is a little lower, and the Ts_wet (21.56 °C) is a little higher by comparing with Figure 7a. This results in a VI-Ts triangle far from the “true” VI-Ts diagram in Figure 7a. The reason of higher Ts_wet and lower Ts_dry in Figure 7c is due to few pixels for determining the dry edge within the range between 0.5 and 1.0 of vegetation cover fraction. This weakness cannot be avoided by the traditional method because it depends on both the full ranges of vegetation cover fraction and land surface moisture. From the comparisons in Figure 7, it is indicated that the proposed method has a similar capability to define the VI-Ts diagram using the coarse-resolution MODIS data just like using the fine-resolution ASTER data.

#### 4.3.2 Comparisons based on MODIS data through the whole year of 2003

**Figure 8.**
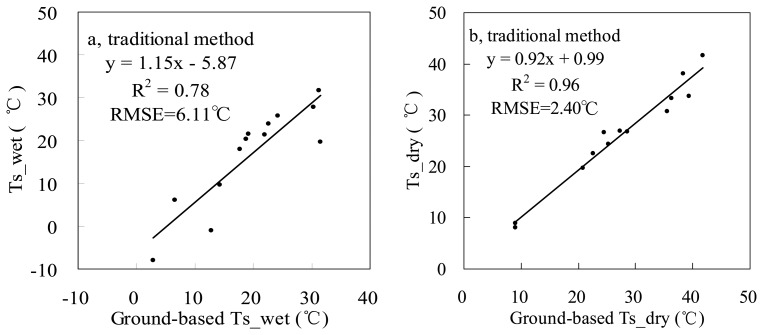
Comparisons of (a) Ts_wet and (b) Ts_dry between from the traditional method based on MODIS data and from ground data across the whole year of 2003.

**Figure 9.**
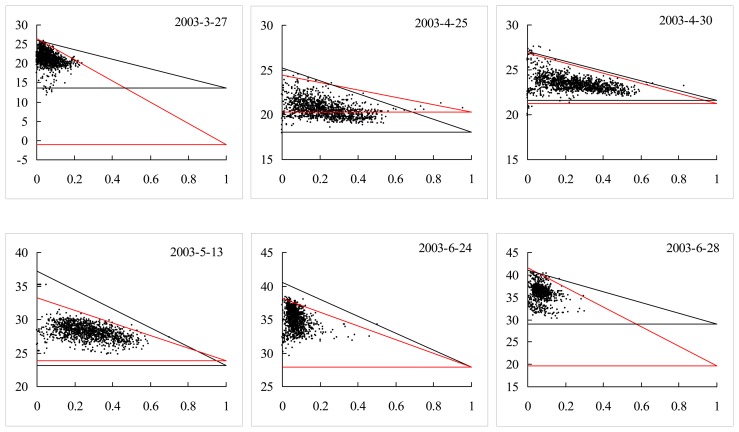

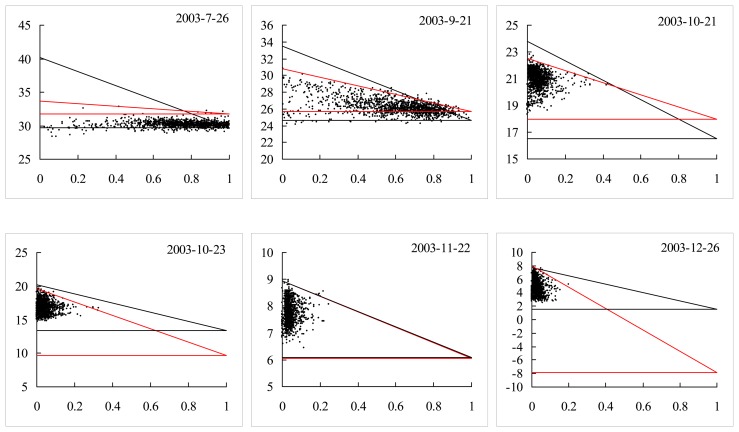
The VI-Ts diagrams defined by the proposed (black) and traditional (red) methods using MODIS data across 2003. The horizontal axis is vegetation cover fraction, and the vertical axis is surface temperature in °C.

**Figure 10.**
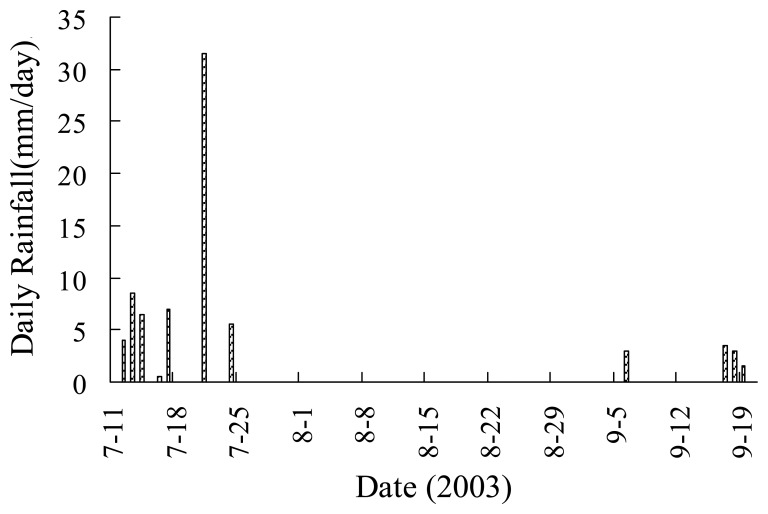
Daily rainfall within the half month just before Jul. 26 and Sep. 21 in 2003.

The proposed and traditional methods were also tested using other 12 MODIS datasets on cloudless days through 2003. Results are shown in Figure 6 and Figure 8 (including results on May 9, 2003), and the VI-Ts diagrams are shown in Figure 9. Both RMSEs of Ts_dry and Ts_wet by the proposed method are less than 1.28 °C, but for the traditional method, the RMSE of Ts_wet is 6.11 °C, and the RMSE of Ts_dry is 2.40 °C. From Figure 9, it is obviously shown that there are larger errors of Ts_dry by the traditional method on Jul. 26 (5.64 °C for absolute bias to the ground-based Ts_dry) and Sep. 21 (4.81 °C for absolute bias to the ground-based Ts_dry). The period from July to September is the rainy season in the NCP. There were 63.5 mm and 11 mm rainfalls in the half month just before Jul. 26 and Sep. 21 in 2003, respectively (Figure 10). Both soil and vegetation had been watered before the EOS-Terra satellite overpassed on Jul. 26 and Sep. 21, so the Ts of bare soil pixels were close to the Ts of vegetation pixels, and the ranges of Ts were only 4.5 °C and 6.68 °C over the study area, respectively. Therefore, it was very difficult to find the true dry point using the traditional method at the 1-km pixel scale in the rainy season, although the concrete, asphalt or some bare soil surfaces within pixels had become dry after the sunrise. In contrast, the proposed method could obtain the dry points with good accuracies based on subpixel information in the rainy season. The absolute biases to the ground-based Ts_dry were reduced to 0.88 °C and 2.09 °C on Jul. 26 and Sep. 21, respectively.

From Figure 9, there are large errors of Ts_wet by the traditional method mainly on Mar. 27 (13.82 °C for absolute bias to the ground-based Ts_wet, before the re-growing of winter wheat), Jun. 28 (11.79 °C for absolute bias to the ground-based Ts_wet, after the harvest of winter wheat) and Dec. 26 (10.71 °C for absolute bias to the ground-based Ts_wet, in winter).The ranges of MODIS-NDVI are all narrower than 0.5 on these three days, so less information on Ts of vegetation pixels contributes to dry edges, and which results in dry edges and Ts_wet with large errors. However, the proposed method can obtain wet points using the minimum *T_veg_* within a sampling window even in the case of narrow range of NDVI. The absolute biases were reduced to 0.75 °C, 2.51 °C and 1.23 °C on Mar. 27, Jun. 28 and Dec. 26, respectively.

## 5. Discussion

### 5.1 Issues about the Bias between MODIS-Ts and ASTER-Ts

MODIS-Ts and ASTER-Ts retrievals were compared by considering the spatial variability of the 11 × 11 aggregated ASTER pixel (90 m) values inside the corresponding MODIS pixel (1 km) in a previous study [27]. 2.5 °C was taken as a given threshold. If the standard deviation of radiometric temperature was larger than 2.5 °C, the MODIS and ASTER pixels were not considered, and then their results showed that selected MODIS-Ts and scaled ASTER-Ts retrievals were in a good agreement. In order to compare all MODIS-Ts and ASTER-Ts over our study area, the threshold of Ts standard deviation was not used to exclude pixels. From Figure 3a, MODIS-Ts is lower than 1 km-ASTER-Ts. The first reason comes from their respective algorithms [22, 23, 27], and the second reason is probably that the spatial variability and scaling issues are not considered [27, 28]. In order to remove these effects, ASTER-Ts was normalized in this study. MODIS-Ts is then relatively consistent with the normalized 1 km-ASTER-Ts (Figure 3b), but MODIS-Ts is still a little lower than 1 km-ASTER-Ts-N, especially for pixels with higher Ts (see points in the upper of scatter plot in Figure 3b). This results in a small shift of dry point in Figure 7. If this shift is igonred, the VI-Ts diagrams are very close between Figure 7a and 7b.

### 5.2 Issues about the Approach of Obtaining T_veg_ and T_soil_

In the proposed method of defining the VI-Ts diagram, the key process is to obtain *T_veg_* and *T_soil_*, and then to determine dry and wet points. In this study, *T_veg_* and *T_soil_* are obtained under the assumption of homogenous land surface moisture for neighboring pixels. Result from [18] show that soil moisture has a spatial correlation within a scale of 6,400 m × 6,400 m in an agricultural landscape. This spatial distribution of land surface moisture is mainly controlled by land cover/use [34]. If the 3 × 3 moving window in Figure 5 covers the same land cover / use, the assumption of homogenous land surface moisture for neighboring pixels is valid. In practice, the 3 × 3 window may covers the boundary between different land covers / uses, for example, the boundary between water and land surfaces, and the boundary between city and surrounding areas. When this case occurs, a land cover / use data such as MOD12 (http://modis-land.gsfc.nasa.gov/landcover.htm) is allowed to exclude pixels whose land types are different to that of the pixel (*i*, *j*) within the 3 × 3 window. When the excluded pixels are more than 3, the values of *T_veg_* and *T_soil_* within the pixel (*i*, *j*) are set as nulls. Actually, there were a lot of 3 × 3 moving windows in the images used in this study, and thus a window with the minimum soil water content can be obtained. It is considered that the surface temperature of soil within this window will be closest to the dry point defined in Figure 1.

### 5.3 Issues about Obtaining Wet and Dry Points

The minimum *T_veg_* (wet point) can be considered as the “true” value of vegetation without water deficit. In this study, we assume that at least vegetation without water deficit can always be found at subpixel scales. Results show that this assumption was valid in our study area. Wet points were obtained with a RMSE error of 1.28 °C through the whole year of 2003. However, true wet points cannot be easily found in some arid regions because well-watered vegetation surface may not exist even at subpixel scales. Wet points will be overestimated in this case.

**Figure 11.**
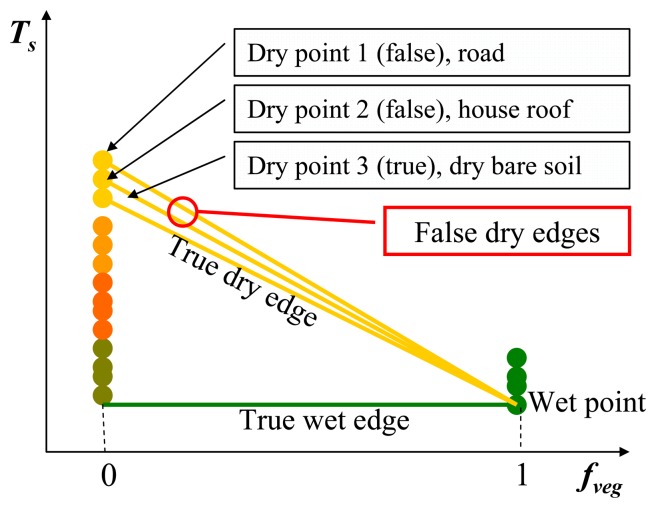
Description of several dry points in the same VI-Ts diagram.

However, *T_soil_* may be not the “true” value of soil but of other components such as roads, house roofs and water surface. In this study, dry point is defined as a surface without vegetation cover and evaporation. Water surface can be excluded because of its low Ts and strong evaporation, but the impervious surfaces such as roads, house roofs satisfy the definition of dry point. It is well known that impervious surfaces have a heating effect through absorbing and holding solar energy, so Ts of them are usually higher than Ts of vegetation, water and general soil. Analysis of ASTER images for examining the relationship between urban thermal features and biophysical descriptors in Indianapolis (Indiana, USA) showed that Ts of impervious surfaces in urban areas were relatively higher than that of soil surface in agricultural areas [35-36]. This was also validated using ASTER images in our study area, and results show that Ts of impervious surfaces in urban areas are 2-6 °C higher than that of soil surface in agricultural areas on May 9, 2003. Further analysis on the VI-Ts diagram from 90 m-ASTER-Ts and 90 m-ASTER-NDVI shows that some scatter points near the position of dry point in the VI-Ts diagram correspond to urban areas. Therefore, there may be several dry points in a VI-Ts diagram, especially in or near urban areas. If several dry points occur in the same VI-Ts diagram (Figure 11), the dry point defined by our proposed method must be the point (dry point 1) with the maximum Ts (e.g.; roads, house roofs), not the true dry point (dry point 3, e.g.; bare soil). In this case, the true dry point will be overestimated. However, this error (RMSE of 1.16 °C for dry point estimations) from this reason is not significant because our study area is an agricultural landscape. In order to identify the true dry point in or near urban areas, the technology of spectral mixture analysis [35-36] is suggested to distinguish bare soil and impervious surfaces (e.g.; roads and house roofs) in further studies. In practices, alternatively, dry point can be determined using the average of several *T_soil_* near the maximum *T_soil_* in order to reduce the effects from impervious surfaces. Sometimes, dry point can also be underestimated in some humid regions because bare soil surface may not exist even at subpixel scales.

### 5.4 Issues about the Definition of the VI-Ts Diagram

To define an ideal VI-Ts diagram, the traditional method requires the full and continuous ranges of land surface moisture and VI [5, 9, 11]. It needs enough heterogeneity of land surface moisture and vegetation cover within a limited sampling window. However, this condition is difficult to meet at coarse-resolution scales such as 1 km scale for MODIS data. For example, narrow ranges of VI resulted in large errors of Ts_wet by the traditional method on Mar. 27, Jun. 28 and Dec. 26 in 2003 in this study. The advantage of our proposed method is independent of the land surface heterogeneity, but only depends on two extreme land surface conditions at subpixel scales, dry bare soil and well-watered vegetation. If dry and wet points can be found within the sampling window, a VI-Ts diagram will be easily defined. This is also a limitation of our proposed method. The proposed method will be helpless to define a VI-Ts diagram if the land cover is entirely homogenous within the sampling window, such as continuous bare soil (e.g.; North African and Mid-Asian deserts) and vegetation (e.g.; tropical and boreal forests) regions. In these regions, all pixels within the sample window will cluster near dry or wet points (no wet point in desert regions, no dry point in forest regions). In applications, if such a cluster occurs in the VI-Ts diagram, it will also contribute to studies about land surface energy balance. The cluster near dry point indicates almost no latent heat partitioned from land surface available energy, while the cluster near wet point indicates almost no sensible heat partitioned from land surface available energy. Even if the land surface is a mixture of bare soil and vegetation, the sampling window should still cover dry bare soil. Vegetation such as forest, grass and crop can be easily found from a mixed land surface. Dry bare soil is also easily found in arid or semiarid areas. However, if the land surface is fully wet across the entire sampling window, such as in humid areas, it is impossible to find dry soil even at subpixel scale. In this case, the VI-Ts scatter plot looks like a horizontal line (no dry point in humid areas), and Ts of all pixels within the sampling window are close to air temperature [10]. Almost all available energy of land surface is transferred to latent heat in such a case. In brief, our proposed method cannot define a triangular VI-Ts diagram without dry and wet points. Our study area is a typical semiarid agricultural landscape (a mixture of bare soil and crop), so our proposed method can obtain dry and wet points with good accuracies through the whole year in this study.

Several previous studies reported that a positive relationship of VI-Ts may occur in low-temperature or low-radiation regions [37-40]. If the slope of dry edge in a VI-Ts diagram is positive, the VI-Ts diagram will not contribute to the estimations of land surface moisture status or evapotranspiration from land surface because there is almost no evapotranspiration in the cases of low-temperature or low-radiation. Thus the positive relationship of VI-Ts was seldom mentioned in applications.

## 6. Conclusions

The traditional method cannot always define a reasonable VI-Ts diagram within a limited sampling window. It is invalid in two cases of rainy season and narrow range of VI. In this study, therefore, a new method was proposed to define a VI-Ts diagram using dry and wet points from the subpixel vegetation and soil information. In this method, a new approach was proposed to obtain *T_veg_* and *T_soil_* within a given pixel by means of neighboring pixels. The VI-Ts diagram defined by the proposed method is close to that defined using the ASTER-Ts of pure vegetation and soil. The proposed method can obtain “true” wet and dry points with respective average accuracies of 1.28 °C and 1.16 °C across the whole year of 2003 in our study area. This indicates that our proposed method can define “true” VI-Ts diagrams in our study area across the whole year, even in two cases of rainy season and narrow range of vegetation index. In applications, two limitations of the proposed method should be noted. A triangular VI-Ts diagram cannot be obtained if either dry point or wet point is absent. The other is that some false dry points may be involved in or near urban areas.

## Figures and Tables

**Table 1.** Statistics of the ASTER and MODIS datasets of Ts and NDVI on May 9 2003.

**Dataset**	**Resolution**	**Size** **(pixel×pixel)**	**Min.**	**Max.**	**Mean**	Stdev*	**Range**
15 m-ASTER-NDVI	15 m	2336×2158	0.03	0.74	0.40	0.11	0.71
MODIS-NDVI	1000 m	35×33	0.07	0.85	0.44	0.09	0.78
90 m-ASTER-Ts (°C)	90 m	390×359	17.85	46.85	25.97	2.29	29.00
1 km-ASTER-Ts (°C)	1000 m	35×33	20.40	34.37	26.19	1.63	13.97
MODIS-Ts (°C)	1000 m	35×33	20.05	27.15	22.81	0.86	7.10

* Stdev is the standard deviation.

**Table 2.** Statistics of MODIS-NDVI and MODIS-Ts on 12 cloud-free days in 2003.

Year/Month/Day	MODIS-NDVI					MODIS-Ts (°C)				
	
	Min.	Max.	Mean	Stdev	Range	Min.	Max.	Mean	Stdev	Range
	
2003/3/27	0.05	0.49	0.3	0.07	0.44	11.79	25.91	21.05	1.85	14.12
2003/4/25	0.1	0.86	0.46	0.11	0.75	17.95	24.49	20.63	0.93	6.54
2003/4/30	0.1	0.81	0.48	0.11	0.7	19.95	27.63	2.42	0.82	7.68
2003/5/13	0.15	0.75	0.5	0.1	0.6	24.79	35.17	28.17	1.19	10.38
2003/6/24	0	0.66	0.31	0.05	0.66	29.61	38.35	35.18	1.46	8.74
2003/6/28	0	0.44	0.32	0.05	0.44	30.29	40.55	36.16	1.58	10.26
2003/7/26	0.18	0.93	0.76	0.11	0.75	28.35	32.85	30.19	0.44	4.5
2003/9/21	0.17	0.87	0.69	0.12	0.7	24.13	3.81	26.23	0.93	6.68
2003/10/21	0.03	0.61	0.29	0.06	0.59	18.35	22.79	20.99	0.68	4.44
2003/10/23	0	0.55	0.27	0.06	0.55	14.67	19.63	16.95	0.94	4.96
2003/11/22	0.01	0.52	0.27	0.05	0.51	6.45	8.97	7.74	0.4	2.52
2003/12/26	0	0.46	0.24	0.05	0.46	2.55	7.89	4.94	1.12	5.34

**Table 3.** R^2^ for linear regression over the 28 pixels × 28 pixels area.

**Year/Month/Day**	**R^2^**	

	**Mean**	**Stdev**
2003/3/27	0.56	0.19
2003/4/25	0.59	0.22
2003/4/30	0.57	0.20
2003/5/9	0.59	0.21
2003/5/13	0.63	0.20
2003/6/24	0.60	0.21
2003/6/28	0.57	0.20
2003/7/26	0.60	0.21
2003/9/21	0.60	0.19
2003/10/21	0.60	0.17
2003/10/23	0.61	0.18
2003/11/22	0.58	0.17
2003/12/26	0.55	0.18

**Table 4.** Comparisons of MODIS-T_veg_ and MODIS-T_soil_ with the normalized 90 m-ASTER-Ts of pure vegetation and soil on May 9 2003.

	**Pixels**	**Mean**	**Stdev**	**Range**	**Min.**	**Max.**
90 m-ASTER-T_veg_-N (°C)	356	20.72	0.68	3.36	19.42	22.78
MODIS-T_veg_ (°C)	356	21.24	1.05	3.41	19.53	22.94
90 m-ASTER-T_soil_-N (°C)	549	24.78	2.05	9.17	20.28	29.45
MODIS-T_soil_ (°C)	549	23.98	1.01	6.38	21.92	28.30
